# Ascorbic Acid Seed Priming Enhances Yield and Related Responses in Broccoli Under Water Deficit Stress

**DOI:** 10.3390/plants15132085

**Published:** 2026-07-04

**Authors:** Vijaya R. Mohan, Lord Abbey, Andrew M. Hammermeister, Mason T. MacDonald

**Affiliations:** Faculty of Agriculture, Dalhousie University, Bible Hill, NS B2N 5E3, Canada; labbey@dal.ca (L.A.); andrew.hammermeister@dal.ca (A.M.H.)

**Keywords:** seed preconditioning, photosynthesis, antioxidant, drought, *Brassica oleracea*, chlorophyll, phenolics, abiotic stress

## Abstract

Drought stress significantly constrains broccoli (*Brassica oleracea* L.) productivity by impairing growth, photosynthesis, and yield. Seed priming with ascorbic acid (AsA) has shown promise in enhancing early seedling performance; however, its effects on head development and yield under water deficit remain limited. This greenhouse pot experiment evaluated four seed treatments: non-primed control, water-primed control, 1 mg L^−1^ AsA, and 10 mg L^−1^ AsA under two irrigation regimes: 100% and 50% field capacity. Growth, physiological traits, biochemical responses, and yield were assessed. AsA priming significantly (*p* < 0.05) enhanced plant height, net photosynthesis, and chlorophyll content under both water regimes. Under 100% FC, water priming significantly increased canopy length, whereas under 50% FC, only AsA priming produced a significant increase relative to the non-primed control (*p* < 0.05). Biochemical responses further showed that 10 mg L^−1^ AsA significantly (*p* < 0.05) increased chlorophyll a and chlorophyll b under 50% FC compared with the non-primed control. Proline accumulation was reduced by 10 mg L^−1^ AsA, but this reduction was significant (*p* < 0.05) only under 100% FC. Under 100% FC, 10 mg L^−1^ AsA significantly (*p* < 0.05) increased total phenolic content compared with the non-primed control. Total flavonoid content was significantly (*p* < 0.05) increased by 1 and 10 mg L^−1^ AsA compared with the control, while both water priming and AsA priming significantly (*p* < 0.05) increased carotenoid content and reduced H_2_O_2_ accumulation relative to the non-primed control, irrespective of watering regime. Total yield per plant, measured on a fresh weight basis, significantly (*p* < 0.05) increased with increasing AsA concentration, with 10 mg L^−1^ AsA enhancing yield by 37.8% relative to the water-primed control and by 70.5% relative to the non-primed control, independent of water regime. Percentage dry weight was unaffected by AsA treatment. Overall, AsA seed priming potentially enhanced physiological resilience and fresh yield of broccoli under water-limited conditions, indicating its potential as a low-cost strategy for drought mitigation.

## 1. Introduction

Broccoli (*Brassica oleracea* L.) belongs to the *Brassicaceae* family, which comprises approximately 351 genera and 3977 species, many of which are economically important [1]. This family includes widely cultivated vegetables such as broccoli, cauliflower, and kale that are valued for their nutritional and health-promoting properties [2]. Among Brassica crops, broccoli has gained substantial attention due to its rich nutritional composition and associated health benefits [3]. It is an excellent source of antioxidants, vitamins C and E, glucosinolates, carotenoids, polyphenols, and minerals, which contribute to immune system support and have been linked to reduced risks of cancer and cardiovascular diseases [2,4,5]. Global population growth and climate change are intensifying pressure on food systems, with food demand projected to increase by 35–56% by 2050 [6,7,8]. Improving agricultural productivity, resource-use efficiency, and innovation is therefore essential to strengthen global food security. Among the various abiotic stresses, drought is considered one of the most damaging, significantly reducing crop performance worldwide [9]. Declining rainfall and the increased frequency of dry spells contribute to water scarcity and the development of drought conditions in North America [10]. Such water-limited conditions reduce crop yield and quality [11,12], emphasizing the need for effective drought mitigation strategies such as improving soil organic matter, adopting water-efficient irrigation systems, cultivating drought-tolerant varieties, and applying seed-based technologies [11,12,13].

Seed priming is a widely used agricultural technique that enhances germination, seedling establishment, plant growth, stress tolerance, and biomass accumulation [14,15]. Seed priming involves incubating seeds with a seed preconditioning agent (SPA) to stimulate early physiological processes that support improved seedling development [16,17]. Mechanistically, priming initiates pre-germinative metabolism, including repair of DNA and cell membranes, activation of antioxidant systems, and partial mobilization of seed reserves, so that primed seeds germinate more rapidly and emerge pre-conditioned to tolerate subsequent stress [15,17]. Hydropriming, the simplest priming method, involves controlled seed hydration with water and may independently improve germination and stress tolerance; therefore, it is commonly included as a control to distinguish the effects of hydration from those of the specific priming agent [17,18]. Several compounds have been identified as effective SPAs, including salicylic acid, acetylsalicylic acid, and glycine betaine, which have shown positive effects on carrot germination [19]. In tomato, 5-hydroxybenzimidazole has been reported to improve yield and stress tolerance [20], while pyroligneous acid has enhanced rice yield [21]. In addition, naturally occurring antioxidants such as lycopene, β-carotene, and ascorbic acid (AsA) have been shown to improve tomato dry mass accumulation and photosynthetic activity when used as seed preconditioning agents [16].

Among these compounds, seed preconditioning with AsA is of particular interest because it is a naturally occurring molecule that is permitted for use in organic agriculture systems [22], in addition to being affordable, accessible, and demonstrating benefits across diverse crops [23]. AsA is commonly known as vitamin C, a water-soluble vitamin. AsA is an antioxidant molecule and a key substrate for reducing oxidative damage in plants [24]. It occurs in all plant cell compartments, including the cell wall. AsA regulates several functions in photosynthesis by acting as an enzyme cofactor, aiding in the synthesis of ethylene, gibberellins, and anthocyanins and playing a role in controlling cell growth [25]. AsA also regulates cell division and growth and is involved in signal transduction in plants [26]. Tomato seeds treated with 1 ppm and 10 ppm AsA exhibited a 40% increase in shoot dry mass, a 50% increase in leaf area, and a 223% rise in photosynthetic rate compared with untreated controls [16]. Similarly, wheat seeds primed with 50 ppm AsA produced approximately 26% higher grain yield, along with moderate improvements in germination [27]. In rice, AsA priming at 10 ppm and 20 ppm increased germination rates and dry mass accumulation [28]. Research shows that AsA priming at 10 ppm was found to significantly enhance shoot biomass, photosynthetic rates, root development, and drought tolerance in broccoli seedlings [29,30]. Additionally, AsA seed priming has been shown to enhance germination performance across multiple broccoli cultivars, resulting in a 19% increase in germination vigor, a 36% increase in seedling weight, a 215% increase in seedling length, and a 214% increase in seedling vigor index, along with a reduction in mean germination time [31].

Across these crops, AsA seed priming has improved germination, growth, photosynthetic performance, and yield in taxonomically diverse species, and in broccoli it has specifically enhanced seedling vigor and drought tolerance. While most studies on AsA seed priming in broccoli have primarily focused on germination and early seedling growth, and to our knowledge none followed AsA-primed broccoli through to marketable head yield under contrasting water regimes; there remains a clear need to evaluate its effects on later developmental stages, particularly broccoli head formation and final yield. Therefore, this study aims to address this knowledge gap by conducting a controlled experiment to assess the impact of ascorbic acid seed priming on broccoli growth and yield under contrasting watering regimes. Because AsA is a potent antioxidant and drought injury results largely from the accumulation of reactive oxygen species [26,32], AsA priming was expected to be most beneficial under water deficit. It was hypothesized that AsA seed priming would enhance drought tolerance in broccoli by improving plant growth and yield and strengthening physiological and biochemical stress defense mechanisms, with greater benefits under water deficit stress (50% field capacity) than under well-watered conditions (100% field capacity).

## 2. Results

### 2.1. Effects of AsA Seed Priming on Broccoli Growth and Morphological Development

AsA seed priming significantly (*p* < 0.05) influenced multiple plant growth and physiological parameters, with effects varying across water regimes and experimental duration (Supplementary Table S1). Plant height, canopy length, leaf area, net photosynthesis rate, and chlorophyll content were greater at 100% FC than at 50% FC indicating that water stress had occurred. Plant height, canopy length, and leaf area were significantly (*p* < 0.05) influenced by AsA seed-priming treatments across the sampling weeks (Table 1). For plant height, treatments did not differ significantly within each sampling week. Across all sampling weeks, the primed treatments had significantly (*p* < 0.05) greater canopy length than the non-primed control, indicating that seed priming improved canopy length. In weeks 1, 3, and 7, the 0 mg L^−1^ treatment was not significantly different from the 1 mg L^−1^ and 10 mg L^−1^ AsA treatments, suggesting that water priming alone produced a similar canopy response to AsA priming during these periods. The clearest separation among primed treatments occurred at week 5, where both 1 mg L^−1^ and 10 mg L^−1^ AsA produced significantly greater canopy length than 0 mg L^−1^. Overall, these results suggest that seed priming increased canopy length, but AsA priming provided only limited additional benefits over water priming, except at week 5. For leaf area, few significant (*p* < 0.05) differences were observed among treatments or across weeks. The 10 mg L^−1^ AsA treatment produced significantly (*p* < 0.05) greater leaf area than both the non-primed and water-primed controls at week 5, indicating an additional AsA effect. At weeks 1 and 7, however, 10 mg L^−1^ AsA did not differ significantly from water priming; therefore, these responses could not be attributed specifically to AsA. Overall, the leaf area response suggests that 10 mg L^−1^ AsA had the clearest effect on leaf expansion, especially at week 5, while treatment effects were otherwise not significant. For plant height, a significant (*p* < 0.05) interaction was observed between water regime and sampling week, indicating that plant height changed differently over time depending on both watering regimes (Supplementary Figure S1).

Leaf area was significantly (*p* < 0.05) influenced by the main effect of water regime (Supplementary Figure S2a). Plants grown under well-watered conditions (100% FC) exhibited significantly greater leaf area compared to drought-stressed plants (50% FC). Transpiration rate was significantly (*p* < 0.05) influenced by the main effect of sampling week (Supplementary Figure S2b). The highest transpiration was observed in week 1, followed by a significant decline in weeks 3 and 5. Transpiration partially recovered by week 7, which was significantly higher than in weeks 3 and 5 but remained lower than in week 1.

**Figure 1:**
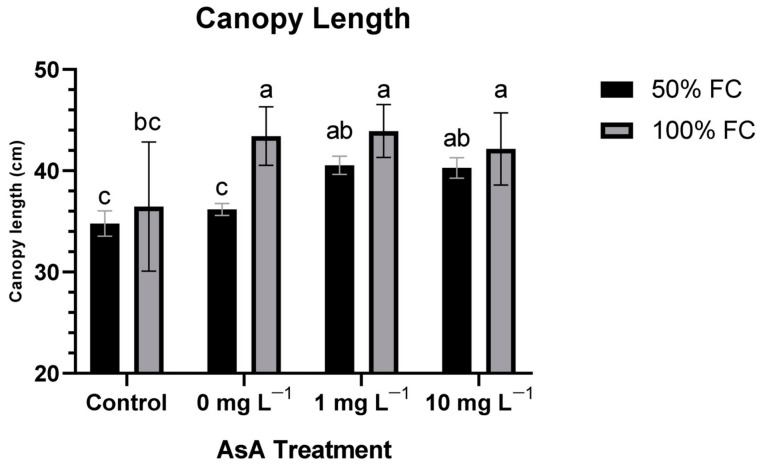
Two-way significant interaction effects of canopy length of broccoli plants measured over 7 weeks after transplanting under well-watered (100% FC) and drought (50% FC) conditions across AsA seed-priming treatments. Means with different letters are significantly different (*p* < 0.05) as determined by Tukey’s honestly significant difference (HSD) test.

A significant (*p* < 0.05) effect of AsA seed-priming treatment was observed for canopy length (Figure 1). Under drought stress (50% FC), canopy length was greater in the AsA-primed treatments than in the non-primed control, with the 1 mg L^−1^ treatment showing the highest response, representing a 23.5% increase over the non-primed control. The 10 mg L^−1^ treatment showed a similar improvement, with a 23.0% increase relative to the non-primed control. Under well-watered conditions (100% FC), canopy length was significantly (*p* < 0.05) greater than under drought stress, and all priming treatments improved canopy length compared with the non-primed control. The 0 mg L^−1^, 1 mg L^−1^, and 10 mg L^−1^ treatments increased canopy length by 22.2%, 22.5%, and 16.0%, respectively, relative to the well-watered control. Although the non-primed control plants consistently showed the lowest canopy length, the response to priming varied with both water regime and AsA concentration.

### 2.2. Effects of AsA Seed Priming on Photosynthetic and Physiological Responses

Net photosynthesis rate and chlorophyll content of broccoli plants were significantly influenced by seed-priming treatment, water regime, and sampling week (Supplementary Table S2).

#### 2.2.1. Net Photosynthetic Rate

**Figure 2:**
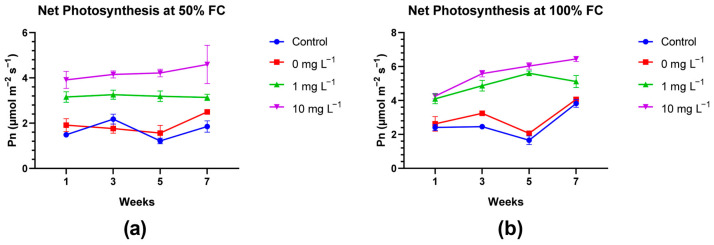
The interaction effects of water regime, and AsA seed-priming treatment on net photosynthetic rate (Pn) under (**a**) 100% and (**b**) 50% field capacity. Weeks 1, 3, and 5 represent the sampling times after transplanting and initiation of the respective watering treatments. Values are presented as least-squares means ± standard error (*n* = 5).

Under water deficit (50% FC) stress, net photosynthesis rate was consistently lowest in the non-primed control and water-primed control across all weeks, ranging from 1.2 to 2.5 µmol m^−2^ s^−1^, whereas AsA priming at 1 mg L^−1^ and 10 mg L^−1^ maintained substantially higher photosynthetic rates throughout the experimental period (3.2 to 4.6 µmol m^−2^ s^−1^), although differences were not always statistically significant as shown in Figure 2a. At week 7, the 10 mg L^−1^ AsA treatment showed the greatest improvement under drought, reaching 4.6 ± 0.4 µmol m^−2^ s^−1^, compared with 1.9 ± 0.4 µmol m^−2^ s^−1^ in the drought-stressed control, representing a 142% increase as shown in Figure 2b. Under well-watered conditions, photosynthesis was also higher in AsA-primed plants, with the 10 mg L^−1^ treatment reaching the highest value at week 7 (6.4 ± 0.4 µmol m^−2^ s^−1^). The largest treatment difference occurred at week 5, where 10 mg L^−1^ AsA increased photosynthesis by approximately 253% compared with the non-primed control. Under water deficit, the non-primed and water-primed controls generally maintained the lowest photosynthetic rates, whereas the AsA treatments produced higher values. Where the AsA treatments differed significantly (*p* < 0.05) from both controls, these responses were interpreted as additional AsA effects. Where AsA and water priming did not differ significantly, no additional effect of AsA beyond controlled hydration was detected.

#### 2.2.2. Chlorophyll Content

**Figure 3:**
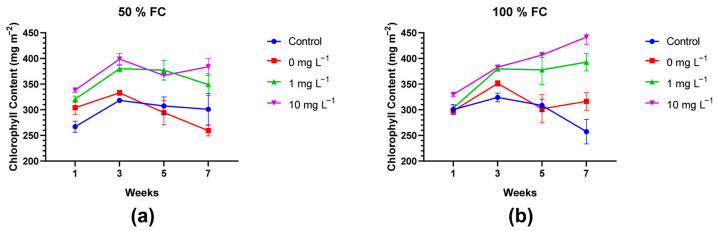
The interaction effects of water regime, and AsA seed-priming treatment on net chlorophyll content (CC) under (**a**) 100% and (**b**) 50% field capacity. Weeks 1, 3, and 5 represent the sampling times after transplanting and initiation of the respective watering treatments. Values are presented as least-squares means ± standard error (*n* = 5).

Similarly, chlorophyll content was generally increased by seed priming, particularly with 1 mg L^−1^ and 10 mg L^−1^ AsA, under both drought and well-watered conditions (Figure 3). Under drought stress, the non-primed control showed lower chlorophyll content across the sampling period (267.0 to 318.2 mg m^−2^), while plants primed with 10 mg L^−1^ AsA maintained comparatively higher values (338.2 to 398.8 mg m^−2^). Water priming alone also increased chlorophyll content at some sampling points, particularly at weeks 1 and 3 under drought stress, compared with the drought-stressed control. Under well-watered conditions, the non-primed control ranged from 257.4 to 324.0 mg m^−2^, while the 10 mg L^−1^ treatment ranged from 329.6 to 441.8 mg m^−2^. However, water-primed control plants also showed higher chlorophyll content than the non-primed control at certain weeks, especially at weeks 3 and 7. Overall, seed priming generally supported chlorophyll retention, however, because water priming produced responses comparable to AsA at some sampling times, the effect was not consistently specific to AsA. The most consistent improvements were observed with 1 mg L^−1^ and 10 mg L^−1^ AsA, while water priming alone also showed some positive effects at specific sampling times.

### 2.3. Effects of AsA Seed Priming on Biochemical and Antioxidant Responses

#### 2.3.1. Chlorophyll a, Chlorophyll b, and Carotenoids

A significant (*p* < 0.05) interaction effect was observed between water regime and AsA seed-priming treatment for chlorophyll a and chlorophyll b (Table 2). In contrast, the interaction effect between water regime and AsA seed-priming treatment was not significant for carotenoids; however, the individual main effects of water regime and AsA treatment were significant (*p* < 0.05) as shown in Table 3. Under 50% FC, chlorophyll a was highest in 1 mg L^−1^, representing a 105.3% increase compared with the non-primed control, followed by 10 mg L^−1^, which showed a 73.8% increase. Chlorophyll b was also highest in 1 mg L^−1^, showing a 76.1% increase over the non-primed control, whereas 0 mg L^−1^ and 10 mg L^−1^ showed increases of 16.0% and 28.2%, respectively. Under 100% FC, chlorophyll a and b followed a similar trend as under 50% FC and were highest in 1 mg L^−1^, representing a 42.6% and 40% increase, respectively, compared with the non-primed control. Carotenoids were lowest in the non-primed control, while 0 mg L^−1^, 1 mg L^−1^, and 10 mg L^−1^ showed increases of 34.2%, 54.0%, and 49.1%, respectively, compared with the non-primed control (Table 3). Although all primed treatments increased carotenoid content relative to the non-primed control, the AsA treatments did not differ significantly from water priming.

#### 2.3.2. Phenolics, Proline, and Flavonoids

A significant (*p* < 0.05) interaction effect was observed between water regime and AsA seed-priming treatment for total phenolics, and proline content (Table 2). In contrast, the interaction effect between water regime and AsA seed-priming treatment was not significant for total flavonoids. Total phenolic content was numerically highest in the 10 mg L^−1^ AsA treatment; however, this increase was not statistically significant compared with the non-primed control, indicating that AsA priming did not produce a clear treatment effect on total phenolics. Proline content was highest in the water-primed control, while 10 mg L^−1^ AsA reduced proline accumulation by 54.7% relative to 0 mg L^−1^. However, the non-primed control did not differ significantly from either the water-primed or AsA-treated plants. Total phenolics were highest in 10 mg L^−1^, representing a 26.3% increase over the non-primed control, while 1 mg L^−1^ showed a 21.5% increase. Proline content was strongly reduced under well-watered conditions, with the lowest values recorded in 1 mg L^−1^ and 10 mg L^−1^, corresponding to 73.3% and 70.1% decreases, respectively, compared with the non-primed control. Under 100% FC, flavonoids were higher compared to 50% FC, representing a 30.1% and 13.7% increase, respectively. Total flavonoids were highest in 10 mg L^−1^, representing a 23.6% increase relative to the non-primed control, followed by 1 mg L^−1^ with a 12.2% increase.

#### 2.3.3. H_2_O_2_ Accumulation

In contrast, the interaction effect between water regime and AsA seed-priming treatment was not significant for H_2_O_2_ production; however, the individual main effects of water regime and AsA treatment were significant (*p* < 0.05) (Supplementary Table S3 and Table 3). H_2_O_2_ production was higher under 50% FC than under 100% FC, representing a 25.6% increase under drought stress. H_2_O_2_ production was highest in the non-primed control, while 10 mg L^−1^ showed significant reductions of 24.3%, respectively, relative to the non-primed control.

### 2.4. Yield Response

**Figure 4:**
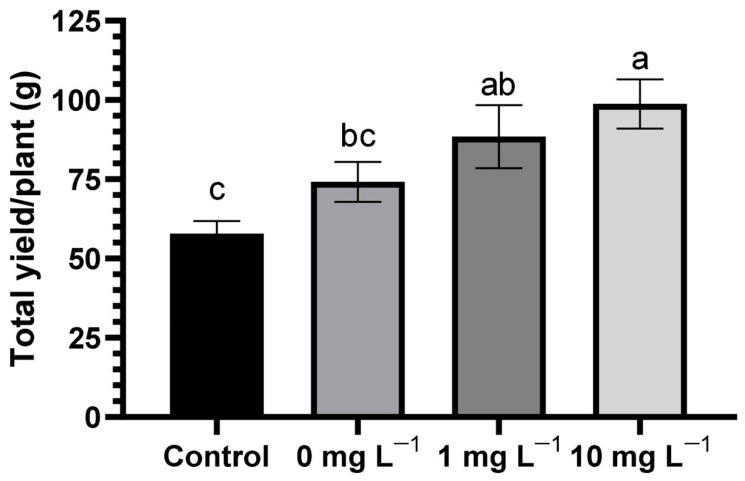
Effects of AsA treatment on total yield per plant (g). Total yield per plant across treatments (Control, 0 mg L^−1^, 1 mg L^−1^, and 10 mg L^−1^). Bars represent means and error bars indicate standard error. Means with different letters are significantly different (*p* < 0.05) as determined by Tukey’s honestly significant difference (HSD) test.

The interaction effect between water regime and AsA seed-priming treatment was not significant (*p* > 0.05) for total yield per plant; however, the individual main effects of water regime and AsA treatment were significant (*p* < 0.05) (Figure 4 and Supplementary Figure S3). Under well-watered conditions (100% FC), total yield was higher than under drought stress (50% FC), showing an approximate 50% increase compared to 50% FC (Supplementary Figure S3). Across AsA treatments, total yield was lowest in the non-primed control, while AsA priming increased yield progressively with increasing concentration (Figure 4). Compared with the non-primed control, total yield increased by 29.1% in 0 mg L^−1^, 52.7% in 1 mg L^−1^, and 70.5% in 10 mg L^−1^. Overall, AsA priming, particularly at 10 mg L^−1^, significantly improved total yield per plant relative to the non-primed control and water-primed control. In contrast, percentage dry weight was not significantly affected by water regime, AsA seed-priming treatment, or their interaction.

## 3. Discussion

The significant effects of seed priming on plant height, canopy length, leaf area, and net photosynthesis indicate that priming generally supported broccoli growth and physiological performance under both water regimes. However, the response was not exclusively due to AsA, as water priming alone also improved several traits compared with the non-primed control, while 10 mg L^−1^ AsA showed potential to further enhance photosynthesis and leaf expansion in some cases. Enhanced net photosynthesis likely increased CO_2_ fixation and carbohydrate production, resulting in greater plant height, canopy length, and leaf area by supplying more assimilates for cell division and elongation, thereby promoting overall vegetative growth [33]. This finding is consistent with previous studies reporting similar improvements in growth responses in both tomato and broccoli [16,29,30].

The interaction among priming treatment and drought was significant (*p* < 0.05) for net photosynthesis, indicating that the effect of AsA priming varied across sampling weeks and between water regimes. Specifically, AsA-primed plants maintained higher net photosynthesis under reduced irrigation at week 7 sampling periods compared with non-primed controls. Although plants subjected to 50% field capacity did not exhibit a marked reduction in transpiration, several biochemical and physiological indicators confirm that they were experiencing stress. Increased H_2_O_2_ and proline accumulation under reduced irrigation reflect activation of oxidative and osmotic stress responses, which are well-established markers of water limitation [32,34]. In addition, the decline in chlorophyll content in the non-primed control further supports the presence of physiological stress. The non-significant decrease in transpiration under water deficit stress suggests only partial stomatal closure, indicating that the imposed treatment represented a moderate water deficit rather than severe drought stress [35,36]. This response pattern is consistent with an early acclimation phase of stress, during which plants detect reduced water availability and initiate biochemical adjustments to enhance water use efficiency without immediately restricting gas exchange. Such a pre-adaptive phase allows maintenance of photosynthetic function while cellular protective mechanisms are activated [36]. Therefore, the 50% FC treatment imposed measurable physiological stress, although not of sufficient severity to trigger pronounced stomatal limitation. The present results indicate that two overlapping processes contributed to broccoli performance, a general hydropriming effect and, for selected traits, an additional AsA effect. Water priming likely acted through controlled imbibition, which initiates pre-germinative metabolism without allowing radicle emergence. During this period, hydration can support membrane reorganization, repair of cellular components, activation of respiration and antioxidant systems, and mobilization of stored reserves [18,37]. These processes may improve subsequent establishment and help explain why water priming performed similarly to AsA priming for canopy length, carotenoid accumulation, H_2_O_2_ regulation, and several other responses. Therefore, where AsA treatments did not differ significantly from the water-primed control, the observed response was interpreted as a general priming effect rather than an AsA-specific effect.

Chlorophyll content in broccoli leaves was significantly (*p* < 0.05) affected by priming treatment, drought stress, and weeks, indicating that AsA seed priming influenced photosynthetic capacity at the pigment level. Primed plants maintained higher chlorophyll concentrations compared to the non-primed control, particularly under drought conditions, suggesting enhanced protection of the photosynthetic apparatus against oxidative damage. These findings are consistent with previous studies on AsA priming in *Chenopodium quinoa* (quinoa), *Brassica oleracea* var. *italica* (broccoli), and *Triticum aestivum* (wheat), where improved chlorophyll retention under water stress was associated with enhanced stress tolerance and sustained photosynthetic performance [29,38,39]. Since chlorophyll is directly associated with light harvesting and carbon fixation efficiency [40], its retention under 50% field capacity reflects improved physiological stability. The preservation of chlorophyll likely contributed to the observed increases in net photosynthesis and vegetative growth parameters, including plant height, canopy length, and leaf area.

A significant (*p* < 0.05) interaction between AsA priming and water regime was observed for chlorophyll a, chlorophyll b, total phenolics, and proline content, indicating that the effect of AsA varies depending on moisture conditions. In contrast, no significant (*p* < 0.05) interaction was detected for carotenoids and H_2_O_2_ production; however, AsA priming independently exerted a significant effect on both parameters. This indicates that carotenoid content and oxidative status were consistently influenced by AsA treatment regardless of water regime. Chlorophyll a is the primary pigment driving photochemical reactions, while chlorophyll b acts as an accessory pigment that broadens light absorption and transfers energy to chlorophyll a, thereby improving photosynthetic efficiency [41]. In this study, AsA priming significantly increased chlorophyll a and chlorophyll b contents, consistent with previous findings reported in broccoli, wheat, quinoa, and mung bean [29,36,38,42]. During photosynthesis, chlorophyll a is essential for electron transport and has to remain stable to support effective photochemical reactions, especially in harsh environments like drought [43]. An adaptive response that promotes photosynthetic efficiency and protects the photosynthetic machinery from stress-induced damage is speculated to be an increase in chlorophyll b [44]. Under drought conditions, increased chlorophyll b enhances light-harvesting capacity, enabling more efficient energy capture and utilization, which can ultimately support vegetative growth and yield [45]. Both water-primed control and AsA priming significantly (*p* < 0.05) increased carotenoid content irrespective of water regime, indicating that its effect on pigment enhancement was consistent under both well-watered and drought conditions. Water priming and AsA priming produced comparable increases in carotenoid content, indicating that carotenoid accumulation was primarily associated with the general priming process. Controlled hydration may have initiated antioxidant and pigment-protective mechanisms before germination, thereby enhancing cellular readiness for subsequent growth [46]. This numerical increase in carotenoids also suggests that AsA may directly stimulate or stabilize carotenoid biosynthesis and thereby preventing the generation of ROS, thus protecting the photosynthetic machinery [47], which is consistent with previous studies on AsA seed priming in Medicago polymorpha, mung bean, quinoa, and broccoli leaves under various stress conditions [29,39,42,48]. Under drought stress, plants often accumulate abscisic acid (ABA), a key hormone involved in stress signaling and stomatal regulation to reduce water loss [49]. Increased carotenoid accumulation during drought may also be associated with ABA-mediated regulation, as ABA can induce the expression of genes involved in carotenoid biosynthesis [50]. Therefore, the enhanced carotenoid levels observed in this study may reflect both the direct influence of AsA on antioxidant metabolism and the indirect regulation of carotenoid synthesis through ABA-related stress signaling pathways. Beyond enhancing stress resilience in broccoli, carotenoids also contribute significantly to human health. These dietary pigments possess strong antioxidant properties that help reduce oxidative stress, support immune function, and lower the risk of chronic diseases, including cardiovascular disorders, certain cancers, and age-related eye conditions such as macular degeneration and cataracts [51].

Proline is an important osmoprotectant that accumulates under water deficit, helping maintain cellular hydration and osmotic balance [52,53]. It also stabilizes membranes and proteins while supporting ROS scavenging and redox homeostasis during stress [54]. The lower proline concentration observed following AsA priming may similarly reflect reduced oxidative stress during early plant establishment. By limiting excessive ROS accumulation, AsA may have reduced stress-induced activation of proline biosynthesis pathways, while potentially favoring proline catabolism through proline dehydrogenase [55,56]. For these reasons, proline is commonly accepted as a viable biochemical biomarker of environmental stress in plants. AsA priming significantly (*p* < 0.05) decreased proline content in broccoli heads, including under drought conditions, which is consistent with findings reported in mungbean following AsA seed priming [42]. Phenolic compounds contribute to plant antioxidant defense by scavenging ROS and limiting cellular damage under drought stress [57,58]. They also enhance the nutritional value of broccoli because their antioxidant and anti-inflammatory properties are associated with reduced risks of several chronic diseases [59]. Flavonoids contribute to plant stress tolerance by scavenging ROS generated during drought-induced oxidative stress [60]. They also enhance the nutritional value of broccoli through their antioxidant, anti-inflammatory, and potential cardioprotective and neuroprotective properties [61]. The observed increase in flavonoid content in broccoli heads under AsA treatment enhanced the nutritional and functional quality of the harvested broccoli heads.

H_2_O_2_ acts as a signaling molecule at moderate concentrations, helping activate protective responses during drought. However, excessive accumulation causes oxidative damage to membranes, chlorophyll, and cellular metabolism [62]. In this study, H_2_O_2_ levels significantly (*p* < 0.05) decreased under AsA priming during water deficit stress, indicating that primed plants were better able to regulate oxidative balance. This suggests that AsA priming enhanced the antioxidant defense system, thereby limiting H_2_O_2_ accumulation and protecting cellular structures under drought conditions. These findings are consistent with previous reports in mungbean where AsA priming reduced oxidative stress by decreasing H_2_O_2_ accumulation [42]. Overall, the reduction in H_2_O_2_ under AsA treatment confirms its role in mitigating drought-induced oxidative damage in broccoli. One possible explanation for the greater chlorophyll response at 1 mg L^−1^ AsA is a hormesis-like effect. Low-level ROS generated under mild stress acts as a transcriptional signal upregulating chlorophyll biosynthesis genes [63,64]. At 1 mg L^−1^ AsA, this signaling may have been sufficiently maintained, whereas the stronger antioxidant effect at 10 mg L^−1^ may have partially reduced ROS-mediated signaling, resulting in a comparatively lower chlorophyll concentration. However, this interpretation remains speculative and would require direct assessment of ROS dynamics and chlorophyll biosynthesis-related gene expression.

**Figure 5:**
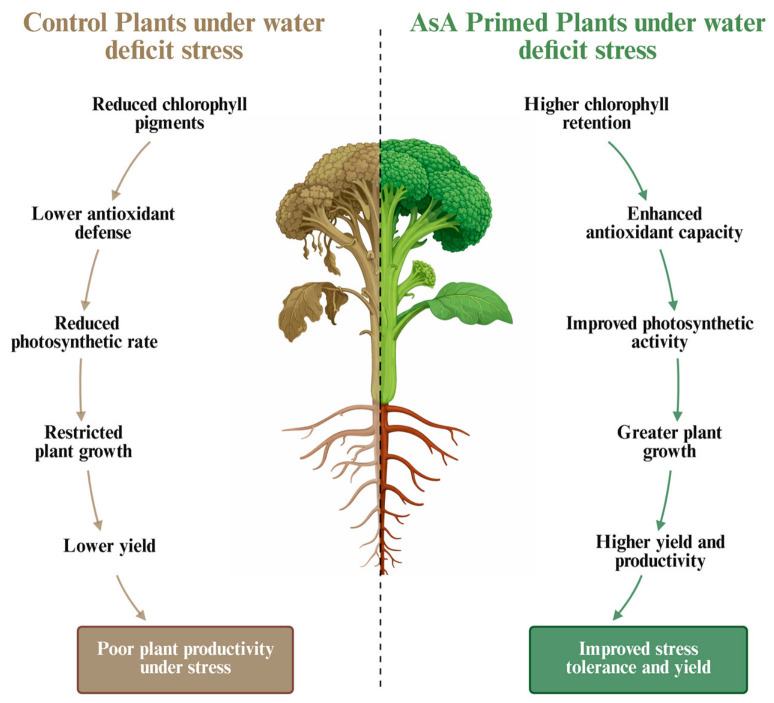
Effects of ascorbic acid (AsA) seed priming on broccoli performance under water deficit stress.

Total broccoli yield was significantly (*p* < 0.05) influenced by both AsA priming and water regime, with the highest yield recorded under 100% field capacity. Water stress is known to reduce nutrient uptake and impair yield [12]; however, among the priming treatments, 10 mg L^−1^ AsA consistently produced the highest yield under both 50% and 100% FC. This response aligns with previous studies reporting yield improvements in rice, wheat, and sunflower following AsA application [65,66,67]. The higher yield observed at 10 mg L^−1^ may be associated with reduced H_2_O_2_ accumulation, improved photosynthetic performance, and enhanced vegetative development. Although this concentration did not maximize all biochemical traits, it produced greater leaf area, which may have increased canopy light interception and whole-plant carbon assimilation [68], thereby compensating for the comparatively lower chlorophyll concentration per unit tissue. Previous studies have shown that moderate reductions in chlorophyll concentration do not necessarily limit canopy photosynthesis, biomass accumulation, or yield when canopy structure and light distribution are improved [69]. Collectively, the greater leaf area, canopy development, and maintenance of photosynthetic activity may have increased assimilate availability for broccoli head development, contributing to the higher yield observed at 10 mg L^−1^ AsA (Figure 5).

The present study indicates that AsA priming can improve broccoli growth, photosynthetic performance, and fresh yield; however, the underlying mechanism remains uncertain. Water priming produced responses comparable to AsA priming for several traits, including carotenoid accumulation and H_2_O_2_ regulation, suggesting that controlled hydration and activation of pre-germinative metabolism contributed substantially to the observed benefits. The additional improvements in selected physiological traits and yield under AsA treatment may reflect early changes in seed redox signaling, enzyme activity, hormone regulation, or gene expression rather than prolonged direct antioxidant scavenging. Because AsA uptake into the seed and molecular responses during imbibition were not measured, future studies should investigate AsA penetration, seed-level redox changes, root architecture, and microbial colonization to clarify how priming produces persistent effects during later plant development. However, these results are derived from a pot study under greenhouse conditions, which may not fully capture field variability in soil, microclimate, and pest pressure. Future research should validate optimal AsA concentrations and priming duration under field conditions and link morpho-physiological responses to detailed biochemical markers, such as chlorophyll, carotenoids, etc., which were measured in this experiment but require further integration with growth and yield outcomes. In addition, future studies should investigate the enzymatic, molecular, and genetic mechanisms underlying AsA seed priming to determine how the observed physiological and biochemical responses are regulated through antioxidant enzyme activity, gene expression, and cellular signaling pathways. Such mechanistic studies would strengthen understanding of the processes responsible for the observed improvements in plant growth, drought tolerance, and yield. Overall, the findings support AsA seed priming as a promising and economically feasible approach for sustaining broccoli production in drought-prone environments.

## 4. Materials and Methods

### 4.1. Experimental Design and AsA Treatment

This experiment was conducted on broccoli as a greenhouse pot study with two experimental factors and one blocking factor. The first experimental factor was seed priming, consisting of four treatments: non-primed seeds (control), water-primed control seeds (0 mg L^−1^), seeds primed with 1 mg L^−1^ AsA, and seeds primed with 10 mg L^−1^ AsA. The second factor was water stress which was applied at two distinct levels as described by [70]. The broccoli cultivar ‘Gypsy’ (Halifax Seed, Halifax, NS, Canada) was selected for this study because it is widely cultivated in North America, particularly in Canada, and is an important commercial variety in regional production systems.

### 4.2. AsA Seed Priming

A 1000 mg L^−1^ stock solution of AsA, with a purity of 99%, in reagent-grade crystalline form from Sigma-Aldrich, Oakville, ON, Canada, was prepared using deionized water. This stock solution was diluted to concentrations of 1 mg L^−1^ and 10 mg L^−1^ with deionized water. All AsA solutions were freshly prepared immediately prior to use, and incubation was conducted in darkness to prevent photooxidation. The pH of the AsA treatment solutions decreased slightly with increasing concentration and was not adjusted: water primed (6.50), 1 mg L^−1^ (6.41), and 10 mg L^−1^ (6.18) reflecting the inherent acidity of ascorbic acid in aqueous solution. Each of the four treatments was transferred to separate 250 mL flasks. Exactly 100 seeds were added to each flask containing 200 mL of AsA solution (or water for the 0 mg L^−1^ treatment) and placed in a G24 Environmental Incubator Shaker (NB Scientific Co., Inc., Woodbridge Township, NJ, USA) set at 150 rpm and 20 °C. After 24 h of incubation in the environmental incubator shaker, the flask contents were poured through a sieve screen, and the seeds were dabbed dry using filter paper. The dried seeds were then directly placed onto moistened filter paper for subsequent analysis. This seed priming procedure was adapted from the method described by [29]. Control seeds did not undergo any priming treatment.

### 4.3. Growing Conditions

Seeds were sown in a greenhouse in March 2025 using two 36-cell seed trays (Feeds N Needs, Truro, NS, Canada), each tray measuring 12.7 × 8.5 × 5.7 cm. Each cell was filled with approximately 7–7.5 g of Pro-Mix Plain BX growing medium (Premier Tech Ltd., Rivière-du-Loup, QC, Canada). The greenhouse maintained a daytime temperature of approximately 25 °C and a nighttime temperature of approximately 18 °C throughout the experimental period with approximately 12 h of natural light. The potting medium, Pro-Mix BX, has a pH range of 5.5–6.5, electrical conductivity of 1.3–2.0 mmhos cm^−1^, air porosity of 17–22% by volume, and water holding capacity of 700–900% by weight. After six weeks, seedlings were transplanted into 12 L pots within the greenhouse, arranged with spacing of 0.6 m × 0.7 m, resulting in a planting density of approximately 2 plants per square meter [71]. Each pot was filled with approximately 1 kg of potting medium. Miracle-Gro^®^ Organics Water Soluble Vegetable & Herb Plant Food (N-P-K 10-1-6) was applied biweekly at a rate of 5 g per plant, directly to the soil near the root zone.

### 4.4. Drought Stress Imposition

All broccoli seedlings were initially cultivated under well-watered conditions for six weeks until transplanting to ensure consistent establishment before water stress was introduced. Subsequently, two irrigation treatments were applied: regular watering (RW), where plants were maintained at 100% field capacity (FC), and drought stress (D), where watering was reduced to 50% FC, simulating drought conditions. Manual watering was performed directly at the base of each plant, avoiding foliage wetting and ensuring precise moisture control. Soil moisture was continuously monitored throughout the experiment using densitometry probe tubes connected to an HH2 Moisture Meter (Delta-T Devices, Cambridge, UK) placed around the roots of plants in both irrigation treatments. Irrigation was applied whenever soil moisture dropped to 40% depletion of the available water, with the volume of water needed to restore the soil to field capacity calculated following the method described by [72]. The blocking factor was based on location within the greenhouse, where the pots were placed in three different positions to account for any environmental variations (e.g., light, temperature, air current) within the greenhouse. To ensure the experiment was sufficiently replicated for statistical analysis, each treatment combination was replicated five times. In total, the experiment involved 40 broccoli plants, with one plant grown per pot.

### 4.5. Measuring Growth and Yield Response

Seed priming was applied once before sowing, using the same priming duration for all primed treatments. Non-destructive growth and physiological measurements were subsequently collected from the same individual plants at one, three, five, and seven weeks after transplanting and initiation of the irrigation treatments. These sampling times were used to determine whether the effects of priming were transient, persistent, or changed as the plants developed and water-deficit stress progressed. Thus, plants compared within each sampling week were of the same chronological age, while sampling week represented successive developmental stages of the same plants. Throughout the growing period, biweekly measurements were taken to assess various plant growth parameters, including plant height, stem girth, canopy length (measured at the widest horizontal aspect), and leaf area [73]. These measurements were conducted using a caliper, meter stick, and measuring tape. For leaf size assessments, largest leaves from each plant were selected from each plant [73]. Harvesting began eight weeks after the plants were transplanted to individual pots. For the primary harvest, all broccoli heads were collected on the same day to maintain consistency in data collection. At harvest, heads were cut at the lowest floret branch contributing to the marketable head to ensure that a consistent amount of stem was retained across plants.

Approximately two to three weeks later, secondary heads from the broccoli plants were gathered. Once all secondary heads had been harvested, the experiment was concluded, and final data analysis commenced. The harvested heads were immediately transported to the lab, cleaned, and measured. Measurements included head counts for each treatment, fresh weight determined using an analytical balance. A representative subsample of known fresh weight was then oven-dried at 80 °C for 2 h and weighed after drying. Dry-matter percentage was calculated as follows (1) [74]:

Dry matter (%) = (dry weight of subsample/fresh weight of subsample) × 100.(1)

Head weights were converted to yield by calculating the total head weight per plant for each treatment. Total yield per plant was determined on a fresh weight basis as the sum of the primary head weight and the combined weight of all secondary heads harvested from the same plant.

### 4.6. Measuring Physiological Response

The chlorophyll concentration was measured using a CCM-300 chlorophyll content meter (Opti Sciences, Hudson, NH, USA). Net photosynthesis (A) and transpiration rate (E), were assessed with an LCi-T Gas Exchange System (ADC Bioscientific, Hoddesdon, UK). Each parameter was measured three times at 30, 60, and 90 s and the reported values represent the average of these readings [30]. These measurements were collected biweekly. Gas exchange measurements were always conducted on a cloudy day, with greenhouse light intensity maintained at 238 ± 5.7 μmol m^−2^ s^−1^ using ambient light, determined from three measurements per block. The temperature was kept at 20 °C, with a relative humidity of 60%, resulting in a vapor pressure deficit of 0.94 kPa.

### 4.7. Measuring Biochemical Response

Harvested head samples from each plant within each treatment were flash-frozen in liquid nitrogen and then ground into a fine powder using a pre-cooled mortar and pestle. The powdered samples were immediately stored at −80 °C for subsequent analyses.

#### 4.7.1. Chlorophyll and Carotenoids

Total chlorophyll a, chlorophyll b, and carotenoid contents were determined following the method described in [75]. Firstly, 0.2 g of ground sample was placed into a sterile 50 mL Falcon tube, and 10 mL of 80% acetone (*v*/*v*) was added. The mixture was vortexed for 1 min and then centrifuged at 12,000× *g* for 15 min. Subsequently, 1 mL of the supernatant was transferred into a cuvette, and absorbance readings were taken at 646.8 nm (A_646.8_) and 663.2 nm (A_663.2_) using a UV-Vis spectrophotometer (Tecan Infinite^®^ M200 PRO, Morrisville, NC, USA), with 80% acetone serving as the blank. Chlorophyll a and chlorophyll b concentrations were then calculated using Equations (2) and (3) below. For the carotenoid concentration, the absorbance was measured at 470 nm and calculated using Equation (4). These concentrations was expressed as μg g^−1^ FW.

Chlorophyll a (μg/g) = 12.25 × A_663.2_ − 2.79 × A_646.8_ (2)

Chlorophyll b (μg/g) = 21.50 × A_646.8_ − 5.1 × A_663.2_ (3)

Carotenoid (μg/g) = (1000 × A_470_ −1.8 × chla−85.02 × chlb)/198, (4)

#### 4.7.2. Total Flavonoids

Total flavonoid content was measured following the method of [76]. To start, 0.2 g of ground sample was homogenized with 2.5 mL of 95% (*v*/*v*) methanol. The mixture was centrifuged at 13,000× *g* for 10 min, and 500 μL of the supernatant was transferred to a new tube. To this mixture, 1.5 mL of 95% (*v*/*v*) methanol, 0.1 mL of 10% aluminum chloride (AlCl_3_), 0.1 mL of 1 M potassium acetate, and 2.8 mL of distilled water were added. The mixture was vortexed and incubated at room temperature for 30 min before measuring absorbance at 415 nm against a blank. Flavonoid content was estimated using a quercetin standard curve and expressed as mg g^−1^ FW.

#### 4.7.3. Total Phenolics

Total phenolic content (TPC) was determined using the Folin–Ciocalteu assay as described by [77]. To start, 0.2 g of ground sample from each treatment was homogenized in 2 mL of ice-cold 95% (*v*/*v*) methanol and incubated in a dark at room temperature for 48 h. The mixture was then centrifuged at 13,000× *g* for 5 min, and 100 μL of the supernatant was transferred to a new microfuge tube. Next, 200 μL of 10% Folin–Ciocalteu reagent was added and vortexed for 5 min. Following this, 800 μL of 700 mM sodium carbonate (Na_2_CO_3_) was added, vortexed for 1 min, and incubated at 25 °C for 2 h. The absorbance of the resulting mixture was measured at 765 nm. Total phenolic content was calculated using a gallic acid equivalents standard curve and expressed as mg gallic acid equivalents per gram of sample.

#### 4.7.4. Proline Content

Proline content was estimated following the method described in [78]. A 0.5 g sample of ground plant material was mixed with 1 mL of 70% ethanol and centrifuged at 12,000× *g* for 15 min at 4 °C. After centrifugation, 500 μL of the supernatant was combined with 1 mL of a reaction mixture containing 1% (*w*/*v*) ninhydrin dissolved in 60% acetic acid (*v*/*v*) and 20% ethanol (*v*/*v*). The tubes were sealed and vortexed for 30 s to ensure thorough mixing, then incubated in a water bath at 95 °C for 30 min. Following incubation, samples were cooled to room temperature, and absorbance was measured at 520 nm using a spectrophotometer. A blank containing only ethanol and the reaction mixture served as the reference. Proline content was calculated using an L-proline standard curve, plotting absorbance against known L-proline concentrations. The total proline content was calculated using Equation (5).

Proline (μmol/g FW) = (Abs_extract_ − blank)/Slope × Vol_extract_/Vol_aliquot_ × 1/FW (5)

where Abs_extract_ is the absorbance of the plant extract, blank is the absorbance of the extraction solution without plant material, the slope is derived from the linear regression of the standard curve, Vol_extract_ is the total volume of extract, Vol_aliquot_ is the volume of extract used in the assay, and FW is the fresh weight of the plant sample.

#### 4.7.5. Hydrogen Peroxide Production

H_2_O_2_ content was quantified following the method of [79]. Briefly, 0.2 g of ground fresh leaf tissue was homogenized in 2 mL of chilled acetone and centrifuged at 10,000× *g* for 10 min. Then, 0.4 mL of titanium (II) chloride and 0.2 mL of 17 M ammonia solution were added to 1 mL of the collected supernatant to form a precipitate. The precipitate was washed five times with acetone through repeated resuspension and subsequently dissolved in 2 mL of 1 M sulfuric acid. Absorbance was recorded at 410 nm against a reagent blank. H_2_O_2_ concentration was determined using a standard calibration curve and expressed as nmol/g.

### 4.8. Statistical Analysis

The effects of AsA seed-priming treatment (4 levels) and water regime (2 levels) on yield and biochemical responses were analyzed using a two-way factorial ANOVA (2 × 4 design). For chlorophyll a, chlorophyll b, total phenolics, and proline, a significant AsA × water regime interaction was detected (*p* < 0.05); therefore, mean separation was performed using Tukey’s multiple comparison test at the 5% significance level, and letter groupings were generated. For yield, carotenoids, total flavonoids, and H_2_O_2_ production, the interaction effect was not significant (*p* > 0.05); therefore, main effects were evaluated using two-way ANOVA, followed by Tukey’s test (α = 0.05) for mean comparisons and letter grouping. Assumptions of normality, homogeneity of variance, and independence were satisfied, and no data transformation was required. All ANOVA analyses were conducted using Minitab (ver. 19.0, Minitab Inc., State College, PA, USA).

Morpho-physiological response variables measured repeatedly at Week 1, Week 3, Week 5, and Week 7 after transplanting were analyzed using repeated measures analysis in SAS [80]. Because the same plants were assessed at successive sampling times, the sampling week was included as the repeated factor in the statistical model. The repeated measures model was used to evaluate the main and interaction effects of AsA treatment, water regime, and sampling week. The most appropriate covariance structure was selected using the Akaike Information Criterion (AIC) [80], and the unstructured covariance matrix provided the best fit. The PROC MIXED procedure in SAS (SAS Institute Inc., Cary, NC, USA) was used to test fixed effects, and LSMEANS was used for multiple comparisons and letter grouping. Model residuals were examined to confirm that assumptions regarding the error terms were met, following the procedures described by [81].

## 5. Conclusions

This study provides potential evidence that AsA seed priming enhances growth performance, physiological function, and yield of broccoli under water deficit conditions, thereby supporting the original hypothesis. Across both irrigation regimes, AsA priming particularly at 10 mg L^−1^ consistently improved some of the key morpho-physiological traits. These enhancements were accompanied by increased accumulation of antioxidant-related compounds and reduced hydrogen peroxide levels, indicating improved oxidative stress regulation. Based on a reagent price of CAD 45 for 25 g of AsA, the cost was approximately CAD 1.80 g^−1^. At the 10 mg L^−1^ concentration used in this study, 100 mL of solution required to treat 50 seeds contained 1 mg of AsA, corresponding to an estimated direct reagent cost of approximately USD 0.0013 per 50 seeds. This calculation indicates that the AsA input itself is inexpensive; however, labor, water, equipment, energy, handling, and commercial-scale processing costs were not assessed. Therefore, further agronomic and economic evaluation is required before its commercial feasibility can be established. Given the low concentration required and minimal application cost, this technique offers substantial potential for adoption in both conventional and organic production systems, particularly in regions prone to water limitation. Future research should focus on field-scale validation across diverse environments and cultivars, as well as integrative approaches such as transcriptomic and metabolomic analyses to better understand the mechanisms driving AsA-mediated stress tolerance. Overall, AsA seed priming emerges as a promising, sustainable strategy to enhance broccoli productivity and resilience under water-limited conditions.

## Figures and Tables

**Table 1:** Interaction effects of AsA seed priming treatments across sampling weeks on plant height, canopy length and leaf area.

Weeks	Treatment	H (cm)	CL (cm)	LA (cm)
**1**	Control	31.2 ± 0.9 c	30.3 ± 1.2 e	108.7 ± 8.7 c
	0 mg L^−1^	35.7 ± 0.9 b	36.9 ± 1.2 c	136.4 ± 8.7 bc
	1 mg L^−1^	35.9 ± 0.9 b	36.1 ± 1.2 cd	162.9 ± 8.7 bc
	10 mg L^−1^	35.4 ± 0.9 bc	35.8 ± 1.2 cd	170.7 ± 8.7 b
**3**	Control	35.4 ± 0.6 bc	33.8 ± 1 d	86.2 ± 8.7 c
	0 mg L^−1^	38.4 ± 0.6 b	39.4 ± 1 bc	95 ± 8.7 c
	1 mg L^−1^	38.7 ± 0.6 b	41.6 ± 1 b	156.3 ± 8.7 bc
	10 mg L^−1^	38.8 ± 0.6 b	41 ± 1 b	147.3 ± 8.7 bc
**5**	Control	38.9 ± 0.6 b	35.8 ± 0.8 cd	112.1 ± 9.9 c
	0 mg L^−1^	39.3 ± 0.6 b	40.6 ± 0.8 b	119.7 ± 9.9 bc
	1 mg L^−1^	40.1 ± 0.6 ab	43.2 ± 0.8 a	150.8 ± 9.9 bc
	10 mg L^−1^	41.2 ± 0.6 ab	43.4 ± 0.8 a	224.7 ± 9.9 a
**7**	Control	40.8 ± 0.7 ab	38.5 ± 1 bc	114 ± 11.1 c
	0 mg L^−1^	40.5 ± 0.7 ab	42.3 ± 1 a	118.5 ± 11.1 bc
	1 mg L^−1^	42.6 ± 0.7 ab	44.1 ± 1 a	152.4 ± 11.1 bc
	10 mg L^−1^	43.7 ± 0.7 a	44.6 ± 1 a	206.8 ± 11.1 ab

H- Plant Height, CL- Canopy length and LA- Leaf Area. Weeks 1, 3, 5 and 7 represent the sampling times after transplanting and the imposition of the 50% and 100% field-capacity treatments. Values represent LS-means ± standard error (*n* = 5). Different letters within a column denote significant differences based on Tukey-adjusted comparisons at *p* < 0.05 following repeated-measures ANOVA. For clarity, where more than three significance group letters occurred, hyphen (-) was used to denote the range of letters. Background shading was used only to improve readability by visually separating week groups and does not indicate statistical significance or additional emphasis.

**Table 2:** Interaction effects showing chlorophyll a, chlorophyll b, total phenolics, and proline concentrations in broccoli heads under four AsA seed-priming treatments in response to different water regimes.

Status	AsA Treatment	Chlorophyll a (µg g^−1^)	Chlorophyll b (µg g^−1^)	Phenolics (mg GAE g^−1^)	Proline (µmol g^−1^)
**50%FC**	Control	4.31 ± 0.21 c	3.01 ± 0.24 d	51.42 ± 3.02 b	5.52 ± 1.06 ab
	0 mg L^−1^	4.32 ± 0.27 c	3.49 ± 0.25 c	54.33 ± 1.57 ab	7.39 ± 0.86 a
	1 mg L^−1^	8.85 ± 0.28 ab	5.30 ± 0.51 ab	57.08 ± 3.03 ab	6.54 ± 1.09 ab
	10 mg L^−1^	7.49 ± 0.27 b	3.86 ± 0.11 bc	61.46 ± 2.80 ab	3.35 ± 0.30 bc
**100%FC**	Control	7.81 ± 0.43 b	4.08 ± 0.18 bc	52.46 ± 2.90 b	4.69 ± 0.46 ab
	0 mg L^−1^	8.00 ± 0.51 b	4.34 ± 0.19 ab	51.12 ± 3.16 b	4.54 ± 0.32 ab
	1 mg L^−1^	11.14 ± 0.79 a	5.71 ± 0.37 a	63.75 ± 5.15 ab	1.25 ± 0.20 c
	10 mg L^−1^	7.80 ± 0.75 b	4.63 ± 0.43 ab	66.26 ± 2.26 a	1.40 ± 0.65 c

Values are expressed as means ± SE of five replicates. Means with different letters within a column denote a significant difference as determined by Tukey’s multiple mean comparison at 5% significance. Columns without letter groupings did not have significant differences between treatments.

**Table 3:** Main effects of AsA seed-priming treatments on carotenoids, total flavonoids, and hydrogen peroxide (H_2_O_2_) production in broccoli heads.

AsA Treatment	Carotenoids (µg g^−1^)	Total Flavonoids (mg g^−1^ FW)	H_2_O_2_ Production (nmol g^−1^)
Control	2.28 ± 0.14 b	69.56 ± 2.66 bc	253.33 ± 13.70 a
0 mg L^−1^	3.06 ± 0.21 a	66.98 ± 2.77 c	225.78 ± 17.03 ab
1 mg L^−1^	3.51 ± 0.22 a	78.06 ± 2.11 ab	210.59 ± 10.48 b
10 mg L^−1^	3.40 ± 0.15 a	85.99 ± 3.41 a	191.82 ± 11.53 b

Values are expressed as means ± SE of five replicates. Means with different letters within a column denote a significant difference as determined by Tukey’s multiple mean comparison at 5% significance. Columns without letter groupings did not have significant differences between treatments.

## Data Availability

The raw data supporting the conclusions of this article will be made available by the authors on reasonable request.

## Supplementary Materials

The following supporting information can be downloaded at: https://www.mdpi.com/article/10.3390/plants15132085/s1, Figure S1: Plant height progression under 50% and 100% field capacity conditions; Figure S2: Main effects of water stress on leaf area and transpiration rate over seven weeks; Figure S3: Total yield per plant under 50% and 100% field capacity; Table S1: ANOVA table of main and interaction effects of treatment, water stress, and week on plant height, canopy length, leaf area, net photosynthesis rate, transpiration, and chlorophyll content; Table S2: Three-way interaction effects of sampling week, water stress, and AsA seed priming on net photosynthesis rate and chlorophyll content; Table S3: Changes in carotenoids, flavonoids, and H_2_O_2_ production in broccoli heads in response to different water levels.
