# Supplementary material for: Ascorbic Acid Seed Priming Enhances Yield and Related Responses in Broccoli Under Water Deficit Stress
Source: Plants (Basel). 2026 Jul 4;15(13):2085. doi: 10.3390/plants15132085 (PMC13364428; doi:10.3390/plants15132085)
Supplement: Supplementary file 1 [file plants-15-02085-s001.zip › plants-4369111-supplementary.pdf]

Supplementary Table S1. ANOVA table of main and interaction effects from treatment (T), water stress (S) and weeks (W) on plant height (H), canopy length (CL), leaf area (LA), net photosynthesis rate (Pn), transpiration (E) and chlorophyll content (CC). Significant ($p < 0.05$) are shown in bold.

Effect	H	CL	LA	Pn	E	CC
**Treatment (T)**	**0.0005**	**<.0001**	**<.0001**	**<.0001**	0.1469	**<.0001**
**Water stress (S)**	**0.0475**	**0.0115**	**0.0057**	**<.0001**	0.8541	**0.0328**
**Weeks (W)**	**<.0001**	**<.0001**	**<.0001**	**<.0001**	**<.0001**	**<.0001**
**T*S**	0.0536	**0.0278**	0.3219	0.0587	0.8867	0.5376
**T*W**	**0.0175**	**0.0037**	**0.0012**	**<.0001**	0.1771	**0.0010**
**S*W**	**0.0296**	0.7019	0.1748	**0.0008**	0.1985	0.3193
**T*S*W**	0.3079	0.1471	0.5958	**0.018**	0.2812	**0.0335**

Supplementary Table S2. Three-way interaction effects of sampling week, water stress, and AsA seed priming (Treatment) on net photosynthesis rate (Pn) and chlorophyll content (CC).

Week	Water stress	Treatment	Pn (µmol m^{-2} s^{-1})	CC (mg m^{-2})
1	50%FC	Control	1.5 ± 0.3 mn	267.0 ± 8.1 bc
		0 mg L^{-1}	1.9 ± 0.3 k–n	304.0 ± 8.1 ab
		1 mg L^{-1}	3.2 ± 0.3 e–l	320.6 ± 8.1 ab
		10 mg L^{-1}	3.9 ± 0.3 d–i	338.2 ± 8.1 ab
	100%FC	Control	2.4 ± 0.3 i–n	300.4 ± 8.1 ab
		0 mg L^{-1}	2.6 ± 0.3 f–n	298.0 ± 8.1 ab
		1 mg L^{-1}	4.1 ± 0.3 c–h	303.2 ± 8.1 ab
		10 mg L^{-1}	4.3 ± 0.3 c–f	329.6 ± 8.1 ab
3	50%FC	Control	2.2 ± 0.2 j–n	318.2 ± 6.1 ab
		0 mg L^{-1}	1.8 ± 0.2 k–n	333.0 ± 6.1 ab
		1 mg L^{-1}	3.3 ± 0.2 d–k	380.0 ± 6.1 a
		10 mg L^{-1}	4.2 ± 0.2 c–g	398.8 ± 6.1 a
	100%FC	Control	2.5 ± 0.2 h–n	324.0 ± 6.1 ab
		0 mg L^{-1}	3.2 ± 0.2 d–k	351.4 ± 6.1 ab
		1 mg L^{-1}	4.9 ± 0.2 a–d	379.6 ± 6.1 a
		10 mg L^{-1}	5.6 ± 0.2 a–c	383.0 ± 6.1 a
5	50%FC	Control	1.2 ± 0.2 n	307.4 ± 19.7 ab
		0 mg L^{-1}	1.6 ± 0.2 l–n	294.4 ± 19.7 ab
		1 mg L^{-1}	3.2 ± 0.2 e–l	377.2 ± 19.7 a
		10 mg L^{-1}	4.2 ± 0.2 c–f	367.0 ± 19.7 a
	100%FC	Control	1.7 ± 0.2 k–n	308.4 ± 19.7 ab
		0 mg L^{-1}	2.1 ± 0.2 k–n	302.0 ± 19.7 ab
		1 mg L^{-1}	5.6 ± 0.2 abc	377.8 ± 19.7 a
		10 mg L^{-1}	6.0 ± 0.2 ab	406.8 ± 19.7 a
7	50%FC	Control	1.9 ± 0.4 k–n	301.0 ± 19.8 ab
		0 mg L^{-1}	2.5 ± 0.4 g–n	259.4 ± 19.8 bc
		1 mg L^{-1}	3.1 ± 0.4 e–m	349.0 ± 19.8 ab
		10 mg L^{-1}	4.6 ± 0.4 b–e	383.6 ± 19.8 a
	100%FC	Control	3.8 ± 0.4 d–j	257.4 ± 19.8 bc
		0 mg L^{-1}	4.1 ± 0.4 c–i	316.4 ± 19.8 ab
		1 mg L^{-1}	5.1 ± 0.4 abc	392.8 ± 19.8 a
		10 mg L^{-1}	6.4 ± 0.4 a	441.8 ± 19.8 a

Weeks 1, 3, 5 and 7 represent the sampling times after transplanting and the imposition of the 50% and 100% field-capacity treatments. Values represent LS-means ± standard error (n = 5). Different letters within a column denote significant differences based on Tukey-adjusted comparisons at $p < 0.05$ following repeated-measures ANOVA. For clarity, where more than three significance group letters occurred, hyphen (–) was used to denote the range of letters.

Supplementary Table S3. Changes in carotenoids, flavonoids, and H_2O_2 production in broccoli heads in response to different water levels.

Status	Carotenoids (μg g^{-1})	Total Flavonoids (mg g^{-1}FW)	H_2O_2 (nmol g^{-1})
**50% FC**	2.66 ± 0.14	70.04 ± 2.18	245.62 ± 11.53
**100% FC**	3.46 ± 0.12	79.62 ± 2.11	195.57 ± 5.59

Values are expressed as means ± SE of five replicates. Means within a column denote a significant difference as determined by Tukey's multiple mean comparison at 5% significance. Columns without letter groupings did not have significant differences between treatments

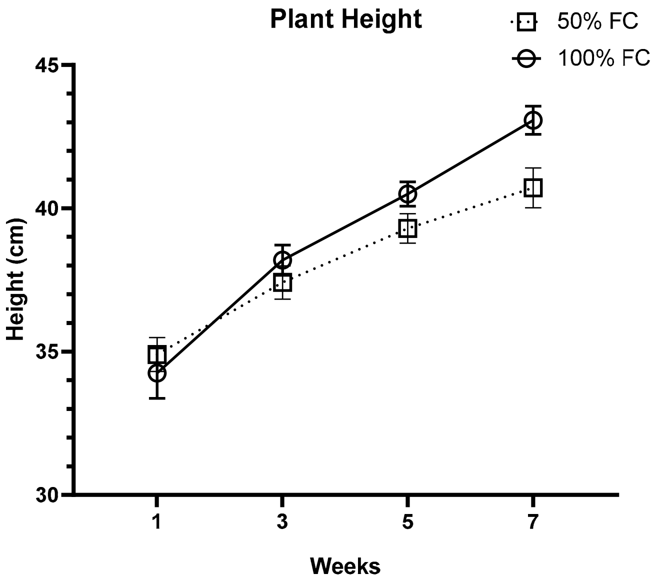

Supplementary Figure S1: Plant height progression under 50% and 100% FC conditions. Each data point represents the mean of 20 plants per treatment, and error bars indicate standard error. Different letters denote significant differences based on Tukey-adjusted comparisons at 5% significance.

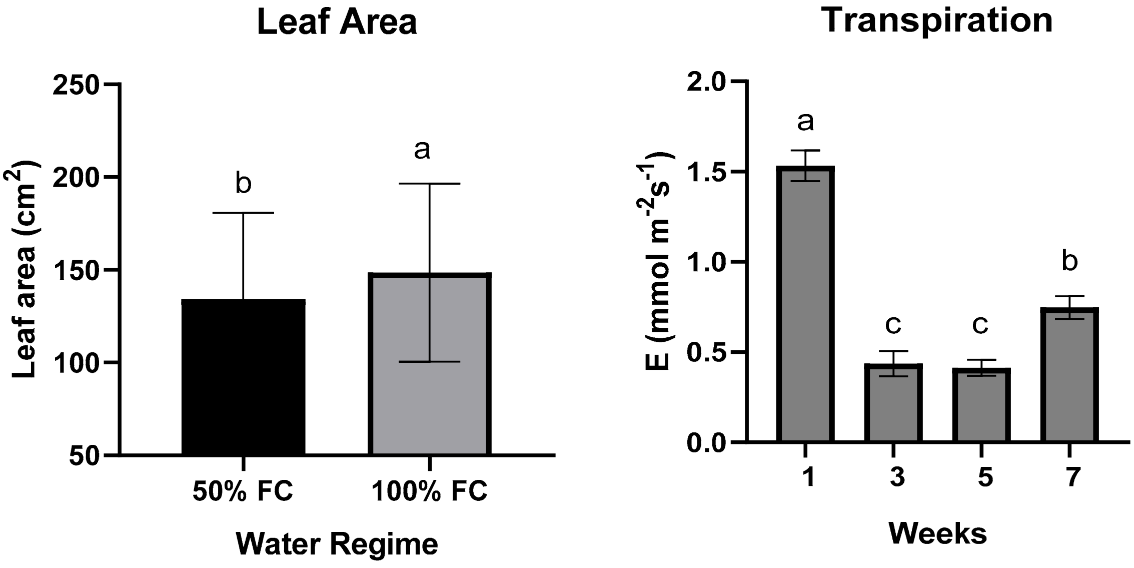

(a) (b)

Supplementary Figure S2. Main effects of (a) Water stress on leaf area measured over seven weeks. Bars represent mean leaf area calculated from 80 plants under 50% and 100% field capacity (FC) conditions. Weeks 1, 3, and 5 represent the sampling times after transplanting and the imposition of the 50% and 100% field-capacity treatments. Error bars indicate standard error and (b) Transpiration rate (E) measured over seven weeks in broccoli plants. Bars represent mean transpiration rate (mmol m^{-2} s^{-1}) at each sampling week calculated from 40 replicates, and error bars indicate standard error.

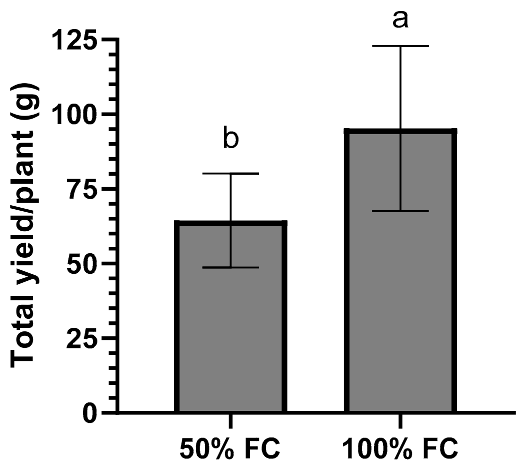

(a)

Supplementary Figure S3. Total yield per plant under 50% field capacity (FC) and 100% FC. Bars represent means and error bars indicate standard error. Means with different letters are significantly different ($p < 0.05$) as determined by Tukey's honestly significant difference (HSD) test.
